# Time and temperature stability of *Tritrichomonas foetus* in phosphate-buffered saline as evaluated by a reverse transcription real-time PCR assay and field analysis

**DOI:** 10.3389/fvets.2023.1101502

**Published:** 2023-03-30

**Authors:** Duan S. Loy, Renata Spuri Gomes, Enakshy Dutta, Bruce W. Brodersen, John Dustin Loy

**Affiliations:** ^1^School of Veterinary Medicine and Biomedical Sciences, Nebraska Veterinary Diagnostic Center, University of Nebraska-Lincoln, Lincoln, NE, United States; ^2^Department of Statistics, University of Nebraska-Lincoln, Lincoln, NE, United States

**Keywords:** time, temperature, RNA stability, PBS, *Tritrichomonas foetus*, direct reverse transcription quantitative real-time PCR

## Abstract

*Tritrichomonas foetus* (TF) is a significant reproductive pathogen of cattle, and sample collection, handling, transport, and testing are significant hurdles to surveillance programs. Recent methods have been developed that allow for the direct detection of TF using a reverse transcription real-time PCR (direct RT-qPCR) approach. To evaluate these methods, a comparative analysis was conducted to assess the technical performance of this assay with a commercially available real-time PCR (qPCR) assay. In addition, the evaluation of two types of collection media (PBS and TF transport tube) was conducted that evaluated sample stability from 0 to 3 days when stored at 4°C or 25°C. Extended incubation times for PBS media were also evaluated (5, 7, and 14 days) at both refrigeration and frozen temperatures to evaluate the effect of extended transport time on samples. Limits of detection (LODs), dynamic range, and RNA stability were assessed using lab-cultured TF spiked into samples of normal bovine smegma collected in PBS or TF transport media, and performance was assessed on field samples collected in parallel. 100% agreement was found between direct RT-qPCR and qPCR at 10 parasites/extraction and a LOD of 1 parasite/extraction. Differences in detection were not observed in either collection media when incubated at either temperatures for up to 3 days of incubation. In addition, the extended incubation experiments indicate that samples containing 10 parasites/extraction can be detected at 4°C for 5 days with a mean Cq 26.34 (95% CI: 23.11–29.58) and detected at −20°C for 7 or 14 days, with a mean Cq 29.55 (95% CI: 27.73–31.37). A significant decrease in detectable RNA was observed in samples containing <10 parasites/extraction at −20°C for 14 days, which should be considered for long-term storage. In summary, direct RT-qPCR was found to be equivalent or superior to qPCR and PBS was not significantly different from TF transport media. The findings of the current study allows for more flexibility during sample collection and transport and ultimately enhancement of TF surveillance programs.

## Introduction

Bovine trichomoniasis is a sexually transmitted disease caused by *Tritrichomonas foetus* (*T. foetu*s). *T. foetus* is a large, pear-shaped, and flagellated protozoan that is an obligate parasite of the bovine reproductive tract (1, 2). Asymptomatic carrier bulls cause infertility in cows and heifers (2). Bovine trichomoniasis has the potential to create significant economic losses in the cattle industry due to embryonic losses and low pregnancy rates (3). The disease has largely been eradicated in Europe due to the widespread adoption of artificial insemination; however, countries such as the United States are still heavily reliant on natural breeding (4, 5). Therefore, many states in the US have import regulations and testing requirements for the movement of breeding animals to control disease transmission and hopefully facilitate eradication. This approach of testing and culling positive bulls has shown some success in individual state programs, such as Wyoming (6). However, the disease remains a widespread challenge, as a recent survey study from 2015 to 2019 conducted in 50 US states revealed 3,817 positive tests in cattle reported in the United States (7). This demonstrates the importance of surveillance testing to enable effective disease control programs.

Current diagnostic methods for bovine trichomoniasis include microscopic examination of incubated pouches (culture), conventional PCR on clinical samples, conventional PCR in combination with enrichment culture, and real-time PCR performed on enrichment culture (8). Both DNA and RNA (cDNA) templates have been used to develop Tritrichomonas foetus PCR-based assays. A recent assay developed by Summarell et al. (9) involves collecting smegma samples in sterile phosphate-buffered saline (PBS) and subsequent amplification of 5.8 rRNA targets for direct reverse transcription quantitative real-time PCR (direct-qPCR). This assay showed enhanced sensitivity and limit of detection compared to culture enrichment followed by real-time PCR of DNA targets (9). Direct-qPCR using PBS instead of a culture medium reduces the cost of testing and simplifies the sample collection process. In addition, the use of PBS may provide less ability for smegma-derived bacteria to overgrow and interfere with PCR testing (10). Even though the increase in abundance of RNA targets likely leads to enhanced performance, other factors influence test performance such as shipping and transport conditions, the incubation period after collecting the smegma sample (11), and the collection method (12). The quality of RNA is essential to allow for reliable results. This is of utmost importance in studies that rely on sample collection in the field or clinical settings because often samples cannot immediately be stored in conditions, such as ultralow temperatures, that prevent RNA degradation, therefore, leading to unreliable results (13). Many factors can compromise the RNA quality affecting its stability and integrity such as inadequate sample handling, prolonged storage, and suboptimal storage conditions (14). In addition, the presence of inhibitors such as blood, urea, salts, and the carry-over of other reagents used during sampling or RNA extraction may also compromise the testing results due to RNA degradation and result in false-negative testing results (15).

Current challenges with trichomoniasis surveillance testing include requirements for rapid transportation to laboratories for testing, incubation of pouches for culture enrichment prior to testing, and requirements for serial sampling in some cases. The ability to directly test clinical samples collected in simple and cost-effective transport media that can be stored for several days during transport would provide enhanced flexibility to surveillance programs.

The objective of this study is to characterize *T. foetus* RNA stability in PBS following time and temperature incubation to simulate field conditions, which included normal smegma to simulate microbial burden during transport. The studies included the stability of RNA in PBS compared with TF media (TF transport tube) for up to 3 days at 4°C and 25°C within a laboratory setting. Another objective was to determine the extended stability of RNA in PBS for up to 5 days at 4°C and to explore the potential effects of freeze/thaw on test performance when held at −20°C for 7 or 14 days. In addition, field samples were collected in parallel to compare test performance. Suitability of samples collected in PBS was also conducted to examine stability and test performance across diagnostic submissions to the Nebraska Veterinary Diagnostic Center (NVDC) for *T. foetus* testing by Cq value comparison.

## Materials and methods

### *T. foetus* culture

A live culture of *T. foetus* strain 17 was obtained from Biomed Diagnostics and was maintained active by the inoculation of a new pouch with one drop (approximately 40 μL) of the active growing culture and incubation (InPouch TF-Bovine, Biomed Diagnostics) at 37°C for 24 h. The organisms were sub-cultured every 3–4 days into fresh pouches to ensure viable cultures. *T. foetus* pouches were enumerated using a hemocytometer. To count the parasites, 15 to 20 μL of the pouch was loaded into the hemocytometer which was placed on the microscope to get the counting grid into focus. The average number of *T. foetus* was 5.64 x 10^6^ cells/ mL.

After enumeration, *T. foetus* culture samples were used to spike 1x phosphate-buffered saline (PBS, Thermo Fisher) and TF media samples containing smegma that was collected from normal, healthy bulls, determined to be free of *T. foetus*. The spiked samples were serial diluted (10-fold) into 100 μL of PBS and 300 μL of TF media (five serial dilutions were made ranging from 1 × 10^5^ to 1 parasite/extraction) and tested in triplicate.

### Sample processing in different transport media

#### TF media samples

*T. foetus* samples were cultured and enumerated as described earlier. Preputial smegma samples were collected from bulls and processed in the laboratory by centrifugation at 2500 × *g* for 3 min and decanted, and the supernatant was discarded. Then, 1 ml of sterile 1x PBS was added to each tube which was vortexed until the pellet was completely re-suspended. For pooled samples, 200 μL of each sample (up to five samples) was transferred to a new transit tube and vortex to mix. At this point, samples were ready to be stored at −20°C or to be extracted.

#### PBS media samples

Samples were vortexed and ready to be stored at −20°C or to be extracted. For pooled samples, the same procedure as the TF media samples was used.

### Experimental design

*T. foetus* culture samples were diluted into 100 μL of PBS media and 100 μL of TF media (TF Transport tube, Biomed Diagnostics) samples containing smegma. Five serial dilutions were made ranging from 1 × 10^5^ to 1 parasite/extraction.

**Figure 1:**
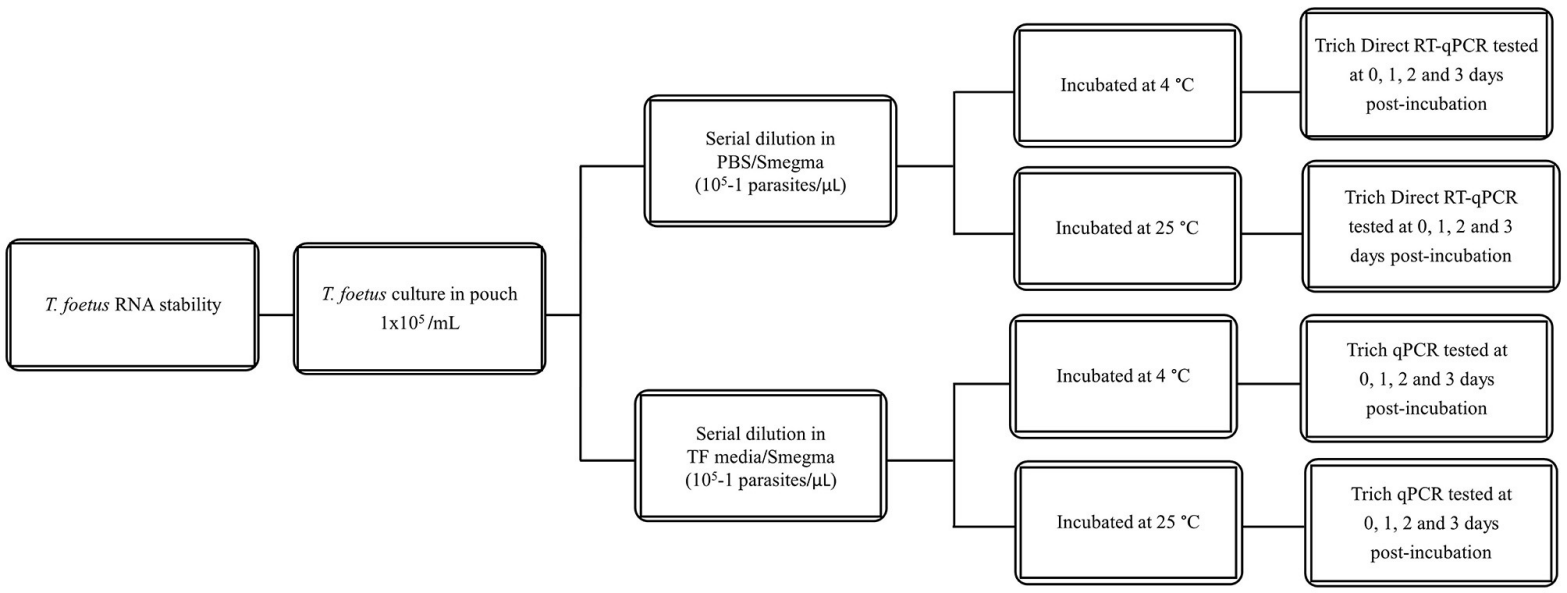
Schematic diagram of stability of *T. foetus* in PBS or TF media up to 3 days post-incubation.

**Figure 2:**
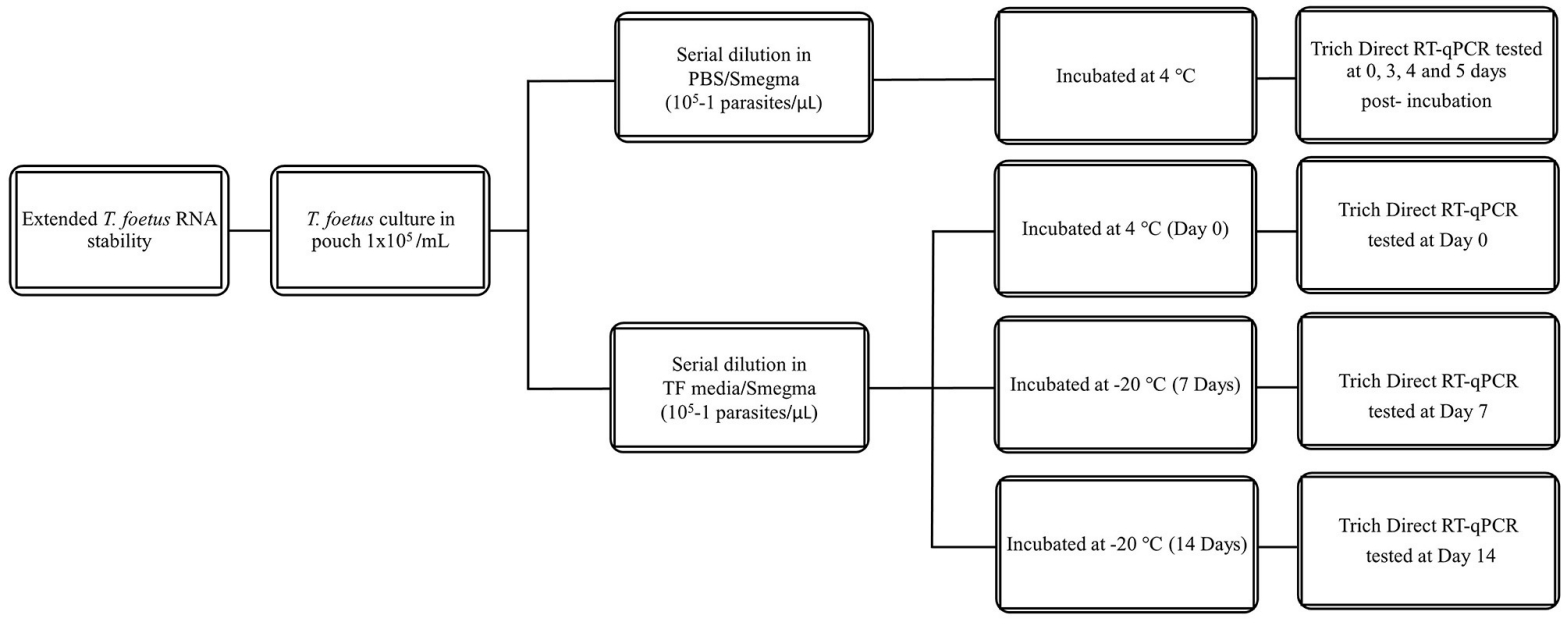
Schematic diagram of extended stability of *T. foetus* in PBS media up to 5, 7, and 14 days post-incubation in different temperatures.

All dilutions were incubated at 4°C or 25°C for the given time points (0–3 days). The experimental design is shown in Figure 1. Then, they were assayed by qPCR and/or Trich direct RT-qPCR. For extended time points, only PBS was evaluated as no differences were observed at early time points in RNA stability in both media. The experimental design of extended stability is shown in Figure 2. Data are represented by three independent experimental replications for all time and temperature combinations.

### Field samples collection

A total of 44 individual and 10 pooled field samples submitted by practitioners to be tested at the Nebraska Veterinary Diagnostic Center (NVDC) were used for field validation of the direct RT-qPCR assay. For each sample, two sets of tubes were submitted which included TF tubes and 1.5 ml 1 × PBS yellow-topped screw cap tubes (Sarstedt^®^, catalog: 60.9921.829). All samples were tested using *T. foetus* qPCR assay, and the Trich direct RT-qPCR assay and results were compared and evaluated. Samples were pooled into five samples, and pooling was processed by laboratory staff. Samples were submitted within 1–5 days post-collection.

Surveillance data were obtained from smegma PBS samples submitted to NVDC between October 2020 and October 2022.

### Real-time PCR

#### *T. foetus* DNA qPCR

For comparison testing, samples were evaluated using a commercial qPCR DNA test kit (VetMAX-Gold Trich Detection Kit, Applied Biosystems/Thermo Fisher Scientific) per manufacturer's instructions, with some minor deviations. Instead of collection in InPouch media and overnight incubation, samples were collected in TF-transit tubes.

Samples were extracted using the manufacturer's provided instructions for DNA isolation (MagMAX^TM^ Pathogen RNA/DNA Kit isolation kit (Applied Biosystems) and KingFisher^TM^ Flex automated magnetic particle processor (Thermo Fisher Scientific). Briefly, 300 μL of the sample was transferred to a 96 deep-well sample plate containing 20 μL of bead mix [10 μL NA binding beads and 10 μL of Lysis/Binding enhancer (Life Technologies)] and 700 μL of lysis binding solution (350 μL of lysis/binding solution concentrate, 2 μL of carrier RNA, 2 μL of Xeno^TM^ DNA control (10,000 copies/μL, Thermo Fisher Scientific), and 350 μL of 100% isopropanol).

The VetMAX-Gold Trich Detection Kit (Trichomonas Foetus DNA Test Kit, Thermo Fisher Scientific) was used for Trich qPCR. The qPCR consisted of 12.5 μL of 2x qPCR Master Mix, 1 μL of *T. foetus* primer/probe mix, 3.5 μL of nuclease-free water, and 8 μL of the nucleic acid template. Thermocycler conditions used were 95°C for 10 min (single cycle), 95°C for 15 s, and 60°C for 60 s (40 cycles; Applied Biosystems 7500 Fast Real-Time System). Samples with a *T. foetus* Cq ≤ 40.0 were considered detected.

#### *T. foetus* direct RT-qPCR

For the RT-PCR, the PCR methods were modified from Ginter Summarell et al. (9). For nucleic acid extraction, slight adjustments were made to the qPCR method, which included an exogenous internal positive control (IPC). Briefly, 100 μL of PBS sample containing smegma was transferred to a 96 deep-well sample plate containing 20 μL of bead mix [10 μL NA binding beads and 10 μL of lysis/binding enhancer (Life Technologies)] and 400 μL of lysis binding solution (200 μL of lysis/binding solution concentrate, 1 μL of carrier RNA, 1 μL of intype^®^ IC-RNA (8 × 10^5^ copies/μL, Indical Bioscience), and 200 μL of 100% isopropanol). Subsequently, the sample plate containing the samples and above reagents, wash plates (300 μL of wash solution 1 and 450 μL of wash solution 2), and elution (90 μL of elution buffer) plates were loaded onto the magnetic particle processor.

The reaction conditions were also slightly modified and consisted of 11.25 μL of nuclease-free water, 6.25 μL of Reliance One-Step Multiplex RT-qPCR Supermix (Bio-Rad), 1.25 μL of Trich Primer/Probe Mix (*T. foetus* primers and probe at a final concentration of 10 μM and 2.5 μM, respectively), 1.25 μL NED IPC Primer/Probe mix (NED primers and probe at a final concentration of 4 μM and 2.5 μM, respectively), and 5 μL of nucleic acid template as shown in Table 1. Thermocycler conditions used were 48°C for 10 min (single cycle), 95°C for 10 min (single cycle), and 95°C for 15 s and 55°C for 45 s (40 cycles; Bio-Rad CFX96^TM^ Real-Time System). Samples with a *T. foetus* Cq ≤ 35.0 and IC-RNA Cq < 40.0 were considered detected.

**Table 1:** Sequence for primers and probes used for Trich direct RT-qPCR.

**Primer/Probe**	**Sequence (5'-3')**	**Length (bp)**	**Tm (°C)**	**References**
**Trich direct RT-qPCR**				
Tfoeq_F2	GAACGTTGCATAATGCGATAAGC	23	55.0	(9)
Tfoeq_R1	AACATATATGCGTGTTCTAGCAAGCT	26	56.6	
Tfoeq_pb1	FAM/ATCTTTGAA/ZEN/TGCACATTGCGCGCC /3IABkFQ/	24	61.3	
**Exogenous internal positive control**				
IC-F	GACCACTACCAGCAGAACAC	20	55.4	(16)
IC-R	CTTGTACAGCTCGTCCATGC	20	56	
IC-pb	NED-AGCACCCAGTCCGCCCTGAGCA/-MGBNFQ/	22	62.3	

FAM, 6-carboxyfluorescein; MGB, minor groove binder; IABkFQ, Iowa Black FQ; NED, yellow fluorophore dye (Applied Biosystems); ZEN, ZEN internal quencher positioned between the ninth (9th) and tenth (10th) nucleotide base in the oligonucleotide sequence and Iowa Black^®^ FQ (3IABkFQ) located at the 3'-end (Integrated DNA Technologies).

### Statistical analysis

The Cq values of direct RT-qPCR and qPCR results were determined using SAS software version 9.4. A linear model was used to first determine if the overall effect of the different samples is significant or not. Furthermore, a *t*-test was used to do the multiple pairwise comparisons using Bonferroni adjustment to compare between samples. If the *p*-values > 0.05, then the samples are similar and produce equivalent outcomes.

## Results

### Limit of detection comparison between Trich direct RT-qPCR and Trich qPCR

A comparative analysis was conducted to assess the limit of detection (LOD) of the Trich direct RT-qPCR with a commercially available real-time PCR assay (qPCR). The performance of the direct RT-qPCR and qPCR was analyzed using spiked serially diluted *T. foetus* organism into PBS or TF media containing smegma ranging from 1 × 10^4^ to 1 parasite/extraction. Three replicates of each dilution were analyzed for the mean Cq value. The results are shown in Table 2. The exogenous internal positive controls were successfully amplified in the reactions for both Trich direct RT-qPCR and qPCR with an average mean Cq of 30.93 ± 0.40 and 32.30 ± 0.13, respectively (data not shown). The observed LOD of Trich direct RT-qPCR was defined at the lowest concentration, which was 100% (three out of three total) at 1 parasite/extraction, whereas the observed LOD of qPCR is 10 parasites/extraction. Trich direct RT-qPCR had improved dynamic range with a LOD of 1 parasite/extraction.

**Table 2:** Limit of detection comparison between Trich direct RT-qPCR and qPCR across three replications.

**PCR**	**Parameter**	**Serial dilutions of** ***T. foetus*** **(Number of parasite/extraction)**					**Observed LOD**
		**10** ^4^	**10** ^3^	**10** ^2^	**10**	**1**	
Trich Direct qRT-PCR (PBS media)	Mean Cq SD	15.05 2.05	18.79 2.17	21.7 1.74	26.62 0.64	32.75 0.88	1
	Number of positive/total	3/3	3/3	3/3	3/3	3/3	
qPCR (TF media)	Mean Cq SD	26.91 0.74	30.66 0.59	33.87 1.37	36.28 1.91	36.67 NA	10
	Number of positive/total	3/3	3/3	3/3	3/3	1/3	

The observed LOD is the lowest concentration where both assays showed 100% positive detection. SD, standard deviation; NA, not applicable.

### Field samples evaluation of PBS and TF transport tube

The evaluation of two types of collection media (PBS and TF transport tube) was conducted by collecting field smegma samples serially, in both media. A total of 44 samples were submitted by veterinarians to NVDC in April–May 2020, with each sample submitted in both media types. Received samples were pooled from the same farm with up to five samples per pool into a total of 10 pools. The result of individual samples (*n* = 44) indicated 100% positive agreement (95% CI: 20.7–100) and 100% negative agreement (95% CI: 91.8.0–100) in field samples collected in parallel for *T. foetu*s testing between direct RT-qPCR and qPCR as shown in Table 3. A similar analysis was conducted with pooled samples (*n* = 10), and the result showed 100% positive agreement (95% CI: 20.7–100) and 100% negative agreement (95% CI: 70.1–100).

**Table 3:** Field samples were collected using PBS and TF transport tubes and validated by Trich direct RT-qPCR and qPCR for both individual and pooled samples.

**Trich qPCR**	**Test result**	**Positive**	**Negative**	**Positive % agreement (CI)**	**Negative % agreement (CI)**
**Individual samples – Trich direct RT-qPCR**					
Trich qPCR	Positive	1	0	100% (20.7–100)	100% (91.8–100)
	Negative	0	43		
**Pooled samples – Trich direct RT-qPCR**					
Trich qPCR	Test Result	Positive	Negative	Positive % agreement (CI)	Negative % agreement (CI)
	Positive	1	0	100% (20.7–100)	100% (70.1–100)
	Negative	0	9		

CI, 95% confidence interval.

### Stability of *T. foetus* in PBS or TF media up to 3 days post-incubation using direct RT-qPCR

**Figure 3:**
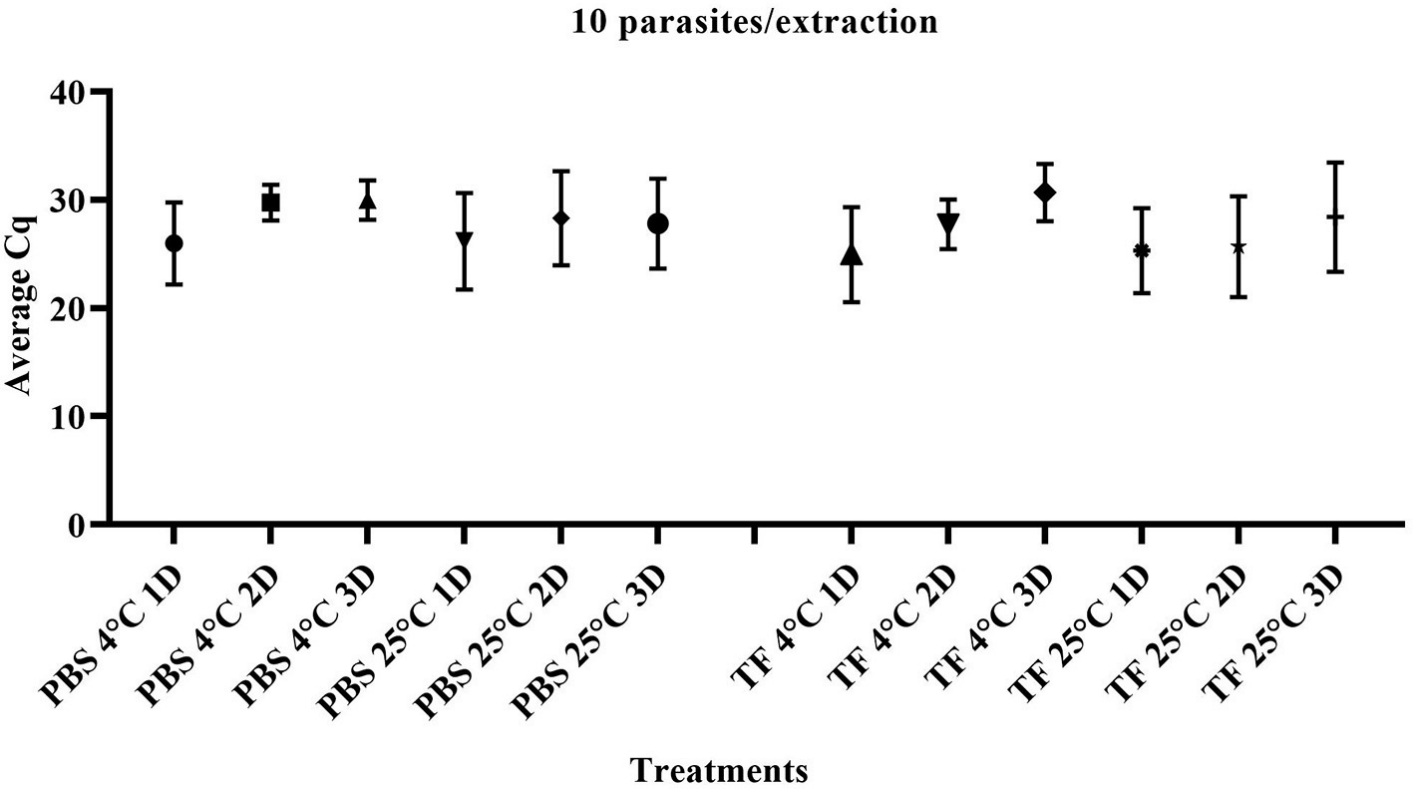
Quantification cycle (Cq) of *T. foetus* RNA in PBS or TF media at 1, 2, and 3 days post-incubation at 4°C and 25°C at 10 parasites/extraction.

To evaluate whether the transport time and temperature affected the outcome of direct RT-qPCR, stability was assessed following incubations ranging from 1 to 3 days along with temperature combinations of 4°C or 25°C in both PBS and TF media. Three replicates of ten-fold serial dilutions ranging from 1 × 10^5^ to 1 parasite/extraction for each treatment were generated. The range and average Cq value of 10 parasites/extraction of samples on days 1–3 are shown in Figure 3. The mean Cq of PCR of all the different dilutions is shown in Supplementary Table 1. A linear model was used to first determine if the overall effect of the media (PBS and TF), temperature (4°C or 25°C), and days (1, 2, and 3) are significantly different using type III fixed effects. Then, multiple pairwise comparisons with a Bonferroni adjustment were conducted to validate that samples produce equivalent results. No significant difference in PBS and TF media in both incubation time and temperature was found (*p* > 0.05). The result indicated that direct RT-qPCR can detect RNA < 10 parasites in either collection media after 3 days when maintained at 4°C or 25°C.

### Extended stability of *T. foetus* RNA in PBS for up to 5 days

To support field applications, RNA stability in PBS up to 5 days post-collection was examined. Given previous results that samples incubated for up to 3 days showed no difference between TF and PBS, these experiments utilized only PBS media and were incubated for up to 5 days. All the pairwise comparisons between day 3 and days 4 and 5 had *p*-values > 0.05, implying that extending PBS at 4°C to days 4 and 5 will produce equivalent results similar to day 3 for all dilutions. However, for 1 parasite/extraction, the upper 95% confidence limit is >35 which approaches the cutoff value except for day 4. The extended incubation experiments also indicate that 10 parasites/extraction can still be detected when maintained at 4°C for 5 days of mean Cq 26.34 (95% CI: 23.11–29.58), while the mean Cq of 1 parasite/extraction was 34.34 (95% CI: 27.51–41.18). The Trich direct RT-qPCR was sufficient to detect RNA of samples containing 10 parasites/ extraction and increased 5 Cq values over the incubation period of 5 days as shown in Table 4. However, this increase in Cq value did not affect overall testing results as it did not exceed the threshold.

**Table 4:** *Tritrichomonas foetus* direct RT-qPCR is shown as mean quantification cycle (Cq) values and an estimated 95% confidence interval (in parentheses).

**Days post collection**	**Serial dilutions of** ***T. foetus*** **(Number of parasite/extraction)**					
	**10** ^5^	**10** ^4^	**10** ^3^	**10** ^2^	**10**	**1**
Day 0	6.31	9.8	14.11	16.96	19.99	31.82
	(4.42–8.20)	(6.59–13.01)	(10.18–18.04)	(13.28–20.64)	(16.76–23.23)	(24.98–38.65)
Day 3	11.86	14.49	17.6	20.97	25.97	30.49
	(9.97–13.75)	(11.28–17.69)	(13.67–21.53)	(17.29–24.65)	(22.74–29.21)	(23.66–37.32)
Day 4	12.75	15.43	19.51	22.81	25.46	25.745
	(10.86–14.64)	(12.22–18.63)	(215.58–23.44)	(19.12–26.48)	(24.22–30.69)	(17.37–34.12)
Day 5	12.765	16.06	18.62	22.4	26.34	34.34
	(10.88–14.65)	(12.85–19.27)	(14.69–22.55)	(18.72–26.08)	(23.11–29.58)	(27.51–41.18)

The positive cutoff value for direct RT-qPCR was Cq ≤ 35.0.

### Extended stability of *T. foetus* RNA in PBS at −20°C

To explore the potential effects of freeze/thaw on test performance and RNA stability, the same experimental design as the previous extended stability study of samples incubated up to 5 days was used; however, in this experiment, samples were tested on day 0 at 4°C and frozen (−20°C) for 7 and 14 days. A pairwise comparison between the samples on day 0 at 4°C vs. days 7 and 14 at −20°C was significant for dilutions 10^4^, 10^3^, 10^2^, and 10 with the 95% confidence limit within the expected range of Cq ≤ 35.0, except for 10^5^ (*p*-value = 0.09) for day 0 at 4°C vs. days 7 at −20°C. For 1 parasite/extraction, although the samples have similar outcomes, the upper 95% confidence limit is >35. This result showed a significant decrease in detectable RNA at < 10 parasites/extraction following incubations held at −20°C for both 7 and 14 days in comparison to day 0 as shown in Table 5. However, the decrease in RNA detection was not sufficient at the 10 parasites/extraction level to affect testing performance as the Cq values did not exceed the threshold to be determined negative.

**Table 5:** *Tritrichomonas foetus* direct RT-qPCR is shown as mean quantitation cycle (Cq) values and an estimated 95% confidence interval (in parentheses).

**Dilution**	**Mean Cq with estimate 95% CI**			**Pairwise comparison (** ***p*** **-value)**	
	**Day 0 at 4**°**C**	**Day 7 at** −**20**°**C**	**Day 14 at** −**20**°**C**	**Day 0 at 4**°**C vs. Day 7 at** −**20**°**C**	**Day 0 at 4**°**C vs. Day 14 at** −**20**°**C**
10^5^	6.31 (1.78–10.83)	13.86 (9.34–18.38)	14.65 (10.96–18.34)	0.09	0.05
10^4^	9.80 (7.78–11.81)	16.99 (14.97–19.00)	19.78 (17.77–21.79)	0.003	0.0004
10^3^	14.11 (12.06–16.15)	21.14 (19.09–23.18)	22.84 (20.80–24.88)	0.003	0.0009
10^2^	16.96 (15.44–18.48)	24.21 (22.69- 25.73)	25.96 (24.44–27.48)	0.001	0.0002
10	19.99 (18.17–21.81)	28.64 (26.82–30.46)	29.55 (27.73–31.37)	0.001	0.0003
1	31.81 (25.61–37.99)	35.03 (28.85–41.20)	34.29 (28.12–40.47)	1	1

The positive cutoff value for Trich direct RT-qPCR was Cq ≤ 35.0. For the pairwise comparisons, the p-value was reported with Bonferroni adjustments.

### Surveillance of trich direct-qPCR in submitted PBS collection tube

**Figure 4:**
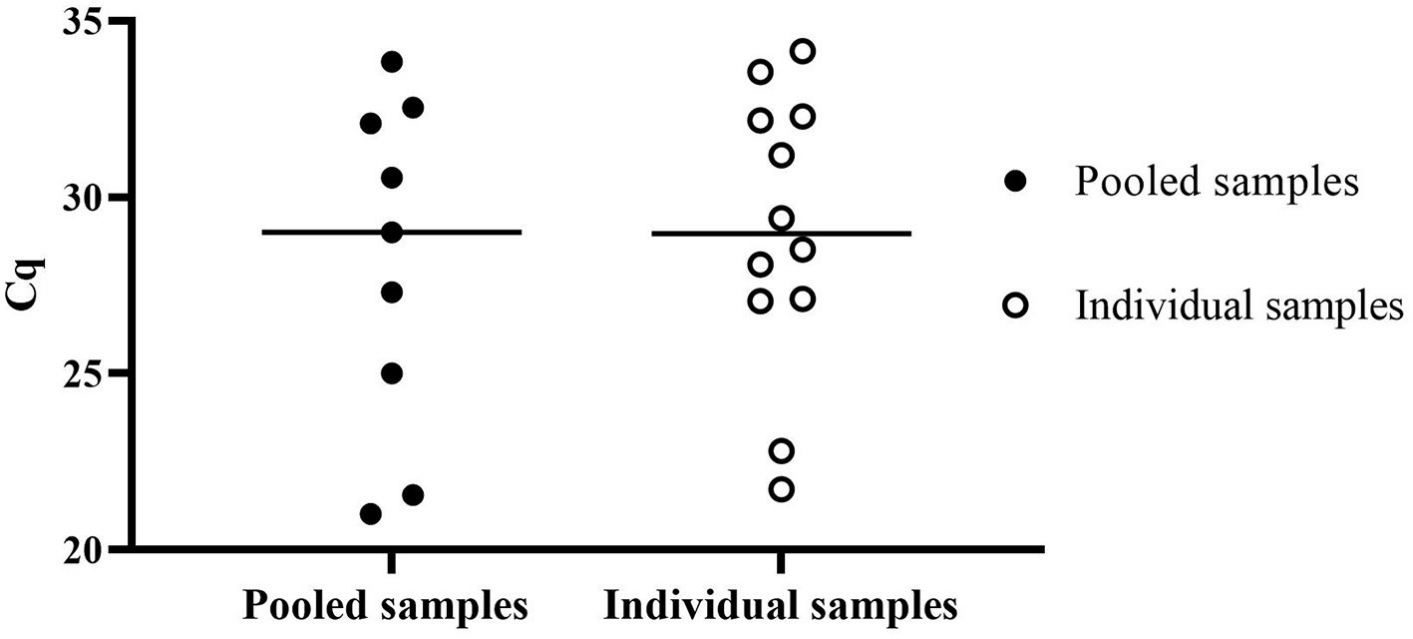
Surveillance data of positive Cq value of *T. foetus* direct RT-qPCR of pooled samples and individual samples submitted from October 2020 to October 2022.

Surveillance data from diagnostic samples were analyzed from submitted smegma samples collected in PBS tubes and submitted to NVDC from October 2020 to October 2022 to assess the performance of direct RT-qPCR. A total of 3,932 animal samples were processed within 5 days post-collection. Twelve of the 776 individual samples were positive, and nine pooled samples of 741 were positive using direct RT-qPCR cutoff Cq ≤ 35.0 as shown in Figure 4. When pooled direct RT-qPCR positive, individual samples were tested to identify the positive animals. The mean Cq value of pooled positive samples is 28.10 ± 4.76, and the mean Cq value of the individual sample is 29.01 ± 3.96.

## Discussion

The detection of *T. foetus* in breeding bulls is an essential step for controlling Trichomonosis which is a risk to herds managed using natural breeding in comparison with artificial insemination (3). The recent study by Ginter Summarell et al. (9) compared the detection of *T. foetus* between culture qPCR (from Trich pouches) and direct RT-qPCR (from smegma samples) assays. The authors' developed a direct RT-qPCR targeting RNA to enhance the sensitivity of the test and demonstrated that direct RT-qPCR presented 100% sensitivity and specificity for smegma samples, while the culture qPCR showed 5% less sensitivity. However, the study did not assess the potential effects of extended times for sample transportation, temperature variation, and pooling of samples. Therefore, our experiments aim to assess the possible effect of RNA detection in different conditions using Trich direct RT-qPCR to enable laboratories to provide evidence-based guidance for sample collection and transport for testing using direct RT-qPCR.

Current methods to identify *T. foetus*-positive bulls include direct examination and culture and molecular methods using conventional and real-time PCR. The latter has been shown to be highly specific and sensitive than culture/microscopic methods and allows for a faster diagnostic turnaround time and elimination of culture medium, making it the recommended technique for the detection of *T. foetus* according to the OIE (8). Several studies have shown improved detection using novel real-time PCR assays (9, 17, 18).

Non-ideal sampling techniques and transport conditions are challenges in the field. These include non-standardized transport media selection, unpredictable time intervals between collection and testing, and seasonal temperature variations among others (19). Bacterial contamination and overgrowth of samples have been shown by Clothier et al. (10) to adversely affect the sensitivity of diagnostics, by interfering with the identification of the parasite by culture and PCR. Two different media formulations were compared: the InPouch^TM^ media and an in-house *T. foetus* culture media modified Diamonds-Plastridge media (DPM), which showed to be more resistant to the effects of bacterial contamination, improving the sensitivity of the diagnostic test (10).

Phosphate-buffered saline (PBS) has the potential for several advantages over existing collection and growth media for TF testing. PBS is readily available in sterile formulations, is inexpensive, does not promote microbial growth, and is shelf stable for long periods of time. In addition, it is clear, allowing for rapid assessment of sample suitability to ensure they are free of blood, feces, or other potential inhibitory substances that can affect PCR performance (20, 21).

Knowing that several contaminants and other issues inherent in field collection can impact RNA stability and subsequently PCR results, in our study, we aimed to simulate these as closely as possible. This included both pooled and individual preputial scrapings that were collected in the field by veterinarians into a 10 mL tube pre-filled with 1.5 mL of PBS and into TF-transit tubes to compare the current qPCR assay with the direct RT-qPCR and to validate the direct RT-qPCR assay. No significant differences were found between the two assays, showing 100% of agreement between them (Table 3). Although we did not assess the impact of bacterial growth on this study, the use of smegma without prior culturing was used to evaluate its potential negative effect on the sensitivity of the assays. The average number of organisms in a sheath scraping from a naturally infected bull is 141 organisms/mL, and it is estimated to be 50 organisms/mL in experimentally infected bulls (22, 23), which is above the limit of detection of 10 parasites/extraction in our study, which used 100 μL of the clinical sample for extraction. However, this comparison may vary depending on the volume of the sample extracted. Furthermore, comparisons based on parasite enumeration should warrant caution, as the enumeration of *T. foetus* by counting has been observed to be inherently inaccurate by others (10). Therefore, given what is known of TF levels in infected animals, the direct RT-qPCR, when collected and shipped using recommended guidelines, should be sensitive enough to routinely detect naturally infected bulls.

RNA-based detection is technically favorable, as there is a much higher abundance of target RNA copies when compared to DNA on a per parasite level, which has been shown to exceed a ratio of 100:1 for some pathogens (24). Several factors have been reported to affect the results of RNA detection (13–15), and given this was the first assay of this type, we conducted a series of studies to evaluate the stability of the target RNA. This was done to simulate a variety of field conditions by the incubation of samples at different temperatures and for various periods of time. We wanted to also evaluate stability in less complex media formulations that reduce the potential for microbial overgrowth such as PBS. We found no significant differences in *T. foetus* RNA stability when spiked-smegma samples in PBS and TF media were incubated at 1, 2, and 3 days at 4°C and 25°C (Figure 3). Similarly, other studies have evaluated the detection of *T. foetus* in different transport conditions using different combinations of environmental temperature and transport time. Clavijo et al. (11) evaluated the effect of three different time durations of transport (24 h, 48 h, and 72 h) and four temperature conditions (4°C, 20°C, 37°C, and 42°C) on culture and qPCR results. They showed that transport temperatures of 4–20°C for 1–3 days were still able to give positive results, although the replication of the parasite was impaired. The authors concluded that temperatures should not be higher than 37° C, and samples should arrive in the laboratory within 24–48 h (11). Another study has shown that elevated temperatures affect TF viability and culture results (19). Additional work has shown that freezing temperatures of more than 1 h result in negative culture results; however, cultures maintained at 22°C or 37°C maintained viability (25).

Although our study did not examine stability at elevated temperatures, we assumed that samples could be readily cooled and shipped at refrigeration temperatures even under field conditions. Given the challenges of transporting samples long distances, we evaluated the effect of extended periods of incubation on the direct RT-qPCR to provide guidance on sample stability on periods >3 days. Our results indicated that samples can be maintained at 4°C for 5 days and −20°C for 14 days. The detection of parasites (10 parasites/extraction) was possible (the average Cq value for 5 days at 4°C was 26.34 (95% CI: 23.11–29.58, Table 4), whereas the average Cq for 14 days at −20°C was 29.55 (95% CI: 27.73–31.37, Table 5). A significant decrease in detectable RNA was shown at < 10 parasites/extraction which may affect test performance. However, in an infected animal, the samples will likely have a parasite burden that exceeds this low level of organisms. As shown in Table 4, the average Cq of 1 parasite/extraction is close to the cutoff Cq of ≤ 35.0. Given this observation, samples that approach or are close to Cq cutoff values, recollection, and testing may be warranted.

In summary, we demonstrated that the direct RT-qPCR is a robust method that allows more flexibility during sample collection and transport and faster turnaround time when compared with other testing approaches. Given these findings, samples of *T. foetus* can still be detected by direct RT-qPCR when held at low temperatures for periods as long as 14 days. These results support recommendations that samples can be held at 4°C for up to 5 days and at −20°C for up to 14 days while maintaining detection limits that are representative of naturally infected bulls.

## Data availability statement

The original contributions presented in the study are included in the article/Supplementary material, further inquiries can be directed to the corresponding author.

## Author contributions

DL, RS, and JL conceptualized the study. DL and RS conducted the experiment and analyzed the data. ED conducted the statistical analysis. DL, BB, and JL finalized the manuscript and analyzed outcomes and discussion. All authors contributed to the article and approved the submitted version.
