# Supplementary material for: Time and temperature stability of Tritrichomonas foetus in phosphate-buffered saline as evaluated by a reverse transcription real-time PCR assay and field analysis
Source: Front Vet Sci. 2023 Mar 30;10:1101502. doi: 10.3389/fvets.2023.1101502 (PMC10098177; doi:10.3389/fvets.2023.1101502)
Supplement: Supplementary file 1 [file Table_1.docx]

| Treatments | Serial dilutions of *T. foetus* (Number of parasite/extraction) | | | | | |
| --- | --- | --- | --- | --- | --- | --- |
|  | 10^5^ | 10^4^ | 10^3^ | 10^2^ | 10 | 1 |
| PBS 4°C_1D | 10.13  (7.29 -12.98) | 14.83  (11.53 - 18.14) | 18.27  (14.78 - 21.75) | 22.07  (18.83 - 25.30) | 25.97  (21.48 - 30.45) | 33.57  (28.74 - 38.39) |
| PBS 4°C_2D | 10.97  (8.12 - 13.81) | 16.2  (12.90 - 19.50) | 19.73  (16.25 - 23.22) | 24.13  (20.90 - 27.37) | 29.73  (25.25 - 34.22) | 35.33  (30.51 - 40.16) |
| PBS 4°C_3D | 11.93  (9.09 - 14.78) | 17.23  (13.93 - 20.54) | 21.53  (18.05 - 25.02) | 26  (22.76 -29.24) | 29.97  (25.48 - 34.45) | 35.8  (30.97 - 40.63) |
| PBS25°C_1D | 10.07  (7.22 - 12.91) | 14.03  (10.73 - 17.34) | 18.57  (15.08 - 22.05) | 21.27  (18.03 - 24.50) | 26.17  (21.68 - 30.65) | 32.13  (27.31 - 36.96) |
| PBS25°C_2D | 10.07  (7.22 - 12.91) | 14.43  (11.13 - 17.74) | 18.6  (15.11 - 22.09) | 22.13  (18.90 - 25.37) | 28.3  (23.82 - 32.78) | 35.8  (29.89 - 41.71) |
| PBS25°C_3D | 11.97  (9.12 - 14.81) | 14.47  (11.16 - 17.77) | 19.6  (16.11 - 23.09) | 23.03  (19.80 - 26.27) | 27.8  (23.32 - 32.28) | 37.35  (31.44 - 43.26) |
| TF 4°C_1D | 10.53  (7.69 - 13.38) | 14.27  (10.96 - 17.57) | 18.07  (14.58 - 21.55) | 21.67  (18.43 - 24.90) | 24.93  (20.45 - 29.42) | 32.07  (27.24 - 36.89) |
| TF 4°C_2D | 9.9  (7.06 - 12.74) | 14.53  (11.23 - 17.84) | 17.97  (14.48 - 21.45) | 20.7  (17.46 - 23.94) | 27.73  (23.25 - 32.22) | 36.1  (31.27 - 40.93) |
| TF 4°C_3D | 10.87  (8.02 - 13.71) | 15.27  (11.96 - 18.57) | 18.73  (15.25 - 22.22) | 23.5  (20.26 - 26.74) | 30.67  (26.18 - 35.15) | 34.9  (30.07 - 39.73) |
| TF25°C_1D | 10.3  (7.46 - 13.14) | 14.13  (10.83 - 17.44) | 17.83  (14.35 - 21.32) | 20.93  (17.70 - 24.17) | 25.3  (20.82 - 29.78) | 35.97  (31.14 - 40.79) |
| TF25°C_2D | 10.2  (7.36 - 13.04) | 13.83  (10.53 - 17.14) | 17.83  (14.35 - 21.32) | 22.1  (18.86 - 25.34) | 25.67  (21.18 - 30.15) | 32.87  (28.04 - 37.69) |
| TF25°C_3D | 10.5  (7.66 - 13.34) | 15.67  (12.36 - 18.97) | 18.5  (15.01 - 21.99) | 21.8  (18.56 - 25.04) | 28.38  (23.90 - 32.86) | 37.1  (32.27 - 41.93) |

**Supplementary Table 1.** Stability of *T. foetus* RNA in PBS or TF media up to 3 days post-incubation*.* Trich direct RT-qPCR are shown as mean quantification cycle (Cq) values and estimate 95% confidence interval (in parentheses). The positive cutoff value for Trich direct RT- qPCR was Cq ≤ 35.0.
